# The Mechanical Separator

**Published:** 1904-05

**Authors:** Garrett Newkirk

**Affiliations:** Los Angeles, Cal.


### THE MECHANICAL SEPARATOR.

By Garrett Newkirk, M.D., Los Angeles, Cal.

It is a truism that free separation, plenty of room, is essential to the proper filling of proximal cavities in teeth and the restoration of natural contour.

This involves two things: first, the obtaining of the space, which may be by slow pressure, necessary in many cases; second, the holding of the space, completely and steadily during the operation. These are two distinct and equally important considerations. In many cases both can be met by the mechanical separator at one sitting; in others, only the latter. There are no rules universal, not subject to exception. Neither is there an "universal" separator which can be depended on to take the place of those adapted to the different regions of the mouth. The "universal" separator is better than none, very often useful, but its principal play is to the pocketbook. It says, "Get me; I will do *all* the work done by the various forms, and *save the cost.*" It appeals to the general desire of mankind to get the most for the least, and something for nothing: an idea full of delusions and snares.

All of the single-bow separators have this objectional feature, that one point crowds into the proximal space precisely where it is not wanted—down upon the gum, also preventing the passage of finishing strips and instruments.

The only complete representation we have of mechanical separation finds expression in the set invented by Dr. S. G. Perry, originally four in number, now six. These cost with box and wrenches $24. About $3 of this amount is for the last named. No need for the case, and any one ought to be able to make his own wrenches. The set proper, less frills, ought to be about $20.

The advantages of the Perry set are that there is something adapted to every place, carefully thought out, and most ingeniously applied. Few are cases indeed where, by reason of unusual forms or relations of the teeth, these separators will not be applicable. As to cost, I can hardly imagine a price on these instruments that would tempt me to get on without them.

Let me enumerate briefly their various spheres of usefulness.

*First:*

### EXAMINATION OF TEETH FOR DECAY.

It is not the rule, I think, that examinations are thorough enough for small cavities and incipient decay upon the proximal surfaces of the teeth near the contact points. Very frequently, owing to the width of their surfaces and their close relations, it is out of the question that an accurate examination can be made without a separation which will admit the rays of light freely to the suspected points. As a rule, I believe, the examiner is disposed to satisfy himself and his patient with the passage of a piece of silk. If this can be done without cutting or a perceptible fraying of the thread he assumes that no decay exists. And the supposition is quite as apt to be wrong as right. No examination has a right to be called complete unless the operator has inspected every danger point upon very tooth, and *knows,* not guesses, the condition of each.

In many cases this cannot be done without the application of the rubber dam and mechanical separation of the proximal surfaces. This applies to the upper teeth from the mesial line to the last intermolar space; and to the lower generally with the exception of the inter-incisal and cuspid.

Whenever there is uncertainty and question, it is easy, only the matter of a little time, to apply the dam to six or more teeth, then go from point to point with the separator, obtaining easily sufficient space for the examination needed without pain and but little discomfort to the patient. If a surface is found which needs but polishing, this may be done at once with oiled strips. If a considerable cavity is discovered, a thin chisel and small bur opens it up for the insertion of gutta-percha as a further separator and temporary stopping. When the operator gets through with his series of explorations, he knows that he has been *thorough* and done the right thing.

*Second*:

IN PREPARATION OF CAVITIES.

Not only is added space necessary for the insertion of fillings, but previous to that for the proper preparation of the cavity. Here the Perry separator will often give it just the room needed with out previous wedging, or it will *hold* and increase the space already obtained. And it holds the teeth *steady*, so that the instrument employed, chisel, excavator or bur, can be used with greater accuracy and less pain, dread or stress on the part of the patient. This, especially in cases where the teeth are not firm, is a very important consideration.

*Third*:

PRELIMINARY TO WEDGING AND TEMPORARY STOPPINGS.

When it is evident that sufficient space cannot be had with the mechanical separator alone, and where faults of previous operations have to be remedied, where a close approximal space needs to be restored, the Perry separator is valuable at the beginning. It is less painful to the patient to open sufficiently for wood or gutta-percha or cotton, than to bear the teasing, gnawing, tormenting piece of a rubber band, so often inserted for that purpose.

And when cavities are opened up sufficiently for gutta-percha stoppings, which are also to act as wedges, the separators are, with me, indispensable in a large proportion of cases.

*Fourth*:

FOR THE INSERTION OF GOLD FILLINGS.

Just as for cavity preparation, the Perry separator holds the teeth in steady position, makes the plugger more accurate and positive, while lessening the discomfort of the patient, always providing, of course, that the screw force is carefully applied and stopped at the proper point. No one is justified, ever, in making his patient really suffer by the use of a mechanical separator. It is not necessary.

In the insertion of gold fillings, the application of the instrument should be gently at first, then slightly increased from time to time as the filling progresses, to the full extension of the contact point and final finish of the same. Then the separator may be employed as a test. Removing the force, allowing the teeth to fall together, one may judge in a few moments, by using floss silk, whether the contour is too full, or just right. If the former, the screw is turned on again, and a little more removed with thin, fine, oiled cuttle-fish strips. The perfect point of contact cannot, as we know, *always* be determined at once. Days afterward, it may be, the discovery will be made by the patient or the operator that the floss cannot be passed at all. The separator can be placed, without the dam, and the tape again passed as before said. How much better this method than the oft-used scratching saw, or the file which invariably cuts away too much, leaving the latter condition worse than the first.

Again, with due deference to certain gentlemen, I will say, for one, that although I do myself occasionally use a matrix for gold fillings, I have just a little more confidence in those built up without it. The separator enables us to dispense with the matrix in most cases where we might be otherwise tempted to employ it.

*Fifth*:

PREPARATORY TO FILLING WITH AMALGAM OR CEMENT.

The separator gives the advantages before described for preparation and initial space. The teeth are held apart and steady up to the moment of applying the matrix, which should, I believe, always be employed in proximo-occlusal fillings of prastic materials. Let me deviate in passing to say that the king of matrices for everyday use is the *Ivory;* for it fits, and demands only one interproximal space for itself. Like every other matrix, it requires occasionally the supplemental wedge, wood or steel, at the cervical border. When relaxed it comes out easily, "leting go" at all points but the "contact," from which it is easily worked free.

For the finishing of amalgam fillings in the contact region, which should never be done until the material is hard, the sepagold.

*Sixth*:

SOME LITTLE POINTS TO REMEMBER.

To adjust the separator easily and deftly takes practice, and the operator must not expect smooth sailing in every case at the first. With the bicuspid and molar forms, the lip is likely to be crowded upward uncomfortably. To relieve this at once, a smooth steel instrument should be inserted at the angle of the mouth and passed gently forward, lifting the lip over.

Whenever the separating points go higher than they should, crowding uncomfortably upon the soft tissues, the screws should be relaxed, the points held in the right position, while pieces of gutta-percha are warmed and crowded under the bows. When these are hard (and the hardening may be hastened with the chip-blower ),th upward movement of the points is prevented and separation proceeded with as usual.

Of course the force must be applied, not all at once, but a little at a time with periods of rest. Only tact and care are necessary. One has to be governed by the conditions present in each case; the sensations of the patient, the mobility of the teeth, and amount of space required.

The screws should be kept carefully oiled, both for ease of working and to prevent their wearing out.

Here, as in relation to all operative procedures, patients have their idiosyncrasies. One will disclaim any sense of discomfort whatever, another suffers more or fears that he will. Once a year, perhaps, a patient will present with whom the separator is impracticable, by reason of unusual sensitiveness of the peridental tissues, or nervous timidity.

Finally, much depends upon our attitude ,when the instrument is first used. We must tell the patient just what we wish to do and why, and what he may expect.

If we explain to the patient, old or young, the necessity of space, the advantages of the separator, often over days of slow wedging; if we assure them that we shall be careful not to carry the pressure beyond the point of reasonable endurance on their part, if we are likewise careful to consult with him or her as to the degree of pressure that may be easily borne, we shall have very little trouble in the use of this most valuable assistant, *the mechanical separator*.

Since writing the above I have had a patient presenting a case where the

### PRACTICAL APPLICATION OF THE SEPARATOR

was well demonstrated.

A lady tourist, from an eastern city, called to have me, if possible, reset a porcelain inlay in a central incisor, distal surface.

It was loose, sure enough, but prevented from falling out by the adjacent lateral, which was a Logan crown.

I said, "The inlay was certainly inserted before the crown in this case."

She replied that such was the case. At first it seemed quite impossible that the inlay could be taken out intact without injury to the cavity margin, or without removing the crown. It occurred to me, however, to see what the Perry separator would do.

In a few minutes time, and with no serious discomfort to the patient, sufficient space had been obtained for the easy removal of the inlay and for its replacement in a manner which I hope will make it permanent.

Of course, one might have succeeded by the use of a wooden wedge, but certainly not with the same positiveness or satisfaction.

NOTE.

Still later, in a conversation with Dr. G. V. Black, he called my attention to the S. S. White "Universal" which I have never tried practically. Dr Black thinks that it *w l* do the work if properly applied in nearly every instance and that it is the most free from objectionable features of any of its class. I have since examined this separator. It seems to represent an attempt and a most ingenious one to carry out the Perry principle in a single instrument. But it is necessarily expensive, larger and more complicated, and it seems to me far preferable that one should shrink his purse, or stretch his credit, just a little more, and possess the real thing; a source of daily comfort and "a joy forever."

Summary.

